# The long and short of it!

**DOI:** 10.1192/bjo.2021.372

**Published:** 2021-06-18

**Authors:** Sidra Chaudhry, Adwaita Ghosh

**Affiliations:** 1 Sheffield Health and Social Care NHS Foundation Trust; 2 Rotherham Doncaster and South Humber NHS Foundation Trust

## Abstract

**Aims:**

The aim of this study was to conduct a literature search on long and short QTc and its implications on prescribing medications.

We also intended to assess the knowledge of psychiatry core trainees in the South Yorkshire region regarding QTc and its implications on prescribing for patients.

**Background:**

The majority of emphasis lies in ensuring the QTc interval is within range for our patients before initiation of psychotropic medication and as part of monitoring during the maintenance phase. The main dread for most psychiatrists is a prolonged QTc interval, however, a short QTc is equally important to identify and manage.

**Method:**

A literature search was performed using the key words “QTc, psychotropics, and ECG”. Results revealed extensive data on long QTc, but very few articles on prescribing psychotropics and short QTc. Most psychotropics are known to prolong QTc interval, which is what clinicians are worried about most when deciding to prescribe medications in mental health services. However, short QTc is also an equally important ECG finding which should not be ignored. We conducted a survey amongst core trainees in the South Yorkshire training scheme to gauge trainees’ knowledge of QTc and its implications when prescribing psychotropic medications. The survey was designed with SurveyMonkey and had seven questions to keep it user friendly.

**Result:**

The survey was distributed to 47 core trainees working in the South Yorkshire region with a response rate of 42.5%. CT1s comprised 30%, CT2s comprised 40% and CT3s comprised 30% of the total number of responders. 60% trainees reported performing and reviewing ECGs as an integral part of their jobs. 50% trainees believed both a short and long QTc interval were life threatening with 50% considering only long QTc as being fatal. 95% of the responders reported not knowing any medications causing QTc shortening; however 100% reported knowing medications causing QTc prolongation.

**Conclusion:**

The results clearly show that we need to increase awareness regarding short QTc interval and its implications on patient health. Review of literature also highlights the challenges in treating patients with QTc abnormalities. In such situations, it's advised to seek advice from Cardiology colleagues to ensure safe and effective patient care. It would also be beneficial to arrange refresher workshops to help psychiatrists brush on their ECG skills.

